# The novel nematicide wact-86 interacts with aldicarb to kill nematodes

**DOI:** 10.1371/journal.pntd.0005502

**Published:** 2017-04-05

**Authors:** Andrew R. Burns, Rachel Bagg, May Yeo, Genna M. Luciani, Michael Schertzberg, Andy G. Fraser, Peter J. Roy

**Affiliations:** 1 The Donnelly Centre for Cellular and Biomolecular Research, University of Toronto, Toronto, Ontario, Canada; 2 Department of Molecular Genetics, University of Toronto, Toronto, Ontario, Canada; 3 Department of Pharmacology and Toxicology, University of Toronto, Toronto, Ontario, Canada; McGill University, CANADA

## Abstract

Parasitic nematodes negatively impact human and animal health worldwide. The market withdrawal of nematicidal agents due to unfavourable toxicities has limited the available treatment options. In principle, co-administering nematicides at lower doses along with molecules that potentiate their activity could mitigate adverse toxicities without compromising efficacy. Here, we screened for new small molecules that interact with aldicarb, which is a highly effective treatment for plant-parasitic nematodes whose toxicity hampers its utility. From our collection of 638 worm-bioactive compounds, we identified 20 molecules that interact positively with aldicarb to either kill or arrest the growth of the model nematode *Caenorhabditis elegans*. We investigated the mechanism of interaction between aldicarb and one of these novel nematicides called wact-86. We found that the carboxylesterase enzyme GES-1 hydrolyzes wact-86, and that the interaction is manifested by aldicarb’s inhibition of wact-86’s metabolism by GES-1. This work demonstrates the utility of *C*. *elegans* as a platform to search for new molecules that can positively interact with industrial nematicides, and provides proof-of-concept for prospective discovery efforts.

## Author summary

Many nematicides that have been used to kill plant and animal parasitic nematodes are being phased out over concerns of toxicity to humans. One potential solution to reduce toxicity is to use the nematicide at a lower concentration in combination with a second compound that together will produce a synergistic killing effect. That is, the use of either molecule alone at low concentrations is non-lethal, but when used together at these same concentrations, the cocktail is lethal. This strategy has two benefits. First, the killing effect is concentrated at the site of use and as the two molecules diffuse from the targeted site, toxicity is negated. Second, less of the toxic molecule is needed and therefore less is dispersed into the environment. Here, we describe our use of a model nematode called *C*. *elegans* to search for molecules that interact with aldicarb, which is one of the nematicides being phased out by environmental agencies. We identified 20 compounds that interact with aldicarb and describe how one of these, called wact-86, functions with aldicarb to kill worms. Our work provides proof-of-principle that *C*. *elegans* is a useful model for identifying compounds that positively interact with industrial nematicides and for understanding the nature of such interactions.

## Introduction

Parasitic nematodes infect more than one billion people worldwide, negatively impacting human health and productivity [1,2]. Dramatic worldwide economic losses are incurred from nematode infections of commercially vital crops and livestock [3–5]. As a result of the growing resistance of nematodes to all of the major anthelmintic classes, the sustained utility of currently available treatments is in doubt, prompting the need for novel interventions [3,4,6,7]. Furthermore, unwanted toxicities associated with otherwise effective anti-nematode treatments has prompted usage restrictions and de-registrations for many nematicides [8,9], providing yet another avenue for attrition. Clearly, novel treatments targeted towards parasitic nematodes are desperately needed.

Aldicarb is one example of a particularly useful anti-nematode agent whose toxicity has limited its utility [10–12]. Aldicarb is a carbamate pesticide that has been used primarily to treat nematode, insect, and mite infections of various economically important crops including cotton and potato [13]. Aldicarb acts similarly to the organophosphate pesticides by inhibiting the enzyme acetylcholinesterase, which hydrolyzes and inactivates acetylcholine, resulting in the accumulation of acetylcholine at synapses [14]. The excess synaptic acetylcholine disrupts the neuromuscular activity of pest organisms, thereby restricting their mobility, arresting growth and impeding host infection. Aldicarb is also able to inhibit cholinesterase activity in non-parasitic animals, which is the mechanism by which it exerts its toxic effects [13,14]. Due to its improper use on watermelon crops in the early 1980s, over 2,000 people in California suffered cholinergic poisoning by aldicarb after eating the contaminated fruit [11,10]. In an effort to avoid additional poisonings, the environmental protection agency in the United States, and other similar agencies around the world, have enacted restrictions and bans on the use of aldicarb [9,15].

In principle, one approach to circumvent the toxicity of aldicarb, or any therapeutic with adverse toxicities, is to combine it with a distinct molecule that can potentiate its effects, such that lower concentrations can be used without compromising efficacy. Indeed, conjunctive therapies have been proposed to mitigate the toxicities of some cancer treatments [16]. In the case of aldicarb, potentiation would ideally not extend beyond the phyla of the parasites it is used to treat, so as to minimize unfavourable toxicity in the host and other non-target organisms.

Through its inhibition of acetylcholinesterase, aldicarb paralyzes and kills the free-living nematode *Caenorhabditis elegans* [17–19]. Thus, one way to find potentiators of aldicarb activity would be to screen chemical libraries, in combination with a sub-lethal dose of aldicarb, for compounds that interact with aldicarb to perturb *C*. *elegans* growth. Unlike many parasitic worms, *C*. *elegans* is readily amenable to high-throughput chemical screens, and it is cheap and easy to culture in the laboratory [20–22]. *C*. *elegans* is not a parasitic nematode, but the majority of commonly used anthelmintics are effective against *C*. *elegans* [23,24], and we and others have shown previously that *C*. *elegans* is a useful model for anthelmintic discovery [25,26]. All of these attributes provide a strong impetus for the use of *C*. *elegans* to screen for new chemical enhancers of aldicarb. Another salient feature of the *C*. *elegans* model is that the mode-of-action of newly discovered bioactive compounds can, in some cases, be determined using straightforward genetic and biochemical approaches [20,25]. An aldicarb interactor screen in *C*. *elegans* has the capacity to identify at least three different classes of compounds: those that potentiate aldicarb activity, those whose activity is potentiated by aldicarb, and those that show mutual potentiation or synergy with aldicarb. Molecules from all three classes hold promise as tools to combat parasitic nematode infection.

Here, we describe our screen of 638 worm-bioactive compounds for those that interact with aldicarb to perturb the growth of *C*. *elegans*. In total, we identified 20 compounds that interact with aldicarb. One of the hits from our screen is the novel worm-active and amide-containing compound wact-86. We use genetic and biochemical methods to demonstrate that wact-86 is hydrolyzed and detoxified in worms by the conserved carboxylesterase enzyme GES-1, and that aldicarb interacts with wact-86 by inhibiting its GES-1-dependent metabolism. Our work builds on ongoing efforts to discover and characterize new anthelmintic synergies [27], and provides proof-of-principle for future screening efforts aimed at identifying and characterizing chemical enhancers of other anti-nematode agents.

## Results

### Aldicarb interacts with the novel worm-bioactive compound wact-86

To find new compounds that interact with aldicarb we screened our in-house library of 638 worm-bioactive compounds [25], which we named the “wactive” library, in combination with a benign 10 μM dose of aldicarb, and assayed for combinations that disrupt the growth of *C*. *elegans* (S1 File; see Methods). As a single agent, aldicarb perturbs worm growth at concentrations above 1 mM (S1 Fig), so our aim was to uncover interactors that increase aldicarb potency by ~100-fold. The wactive library was screened in liquid media at a concentration of 1.5 μM–a condition where worm growth is indistinguishable from the solvent control for 95% of the compounds in the library (S1 File). Our screen identified 20 wactive compounds that perturb worm growth in combination with aldicarb, but are innocuous as single agents at the screening concentration (S2 Fig). The structures of the 20 compounds identified from our screen are shown in S3 Fig.

**Fig 1:**
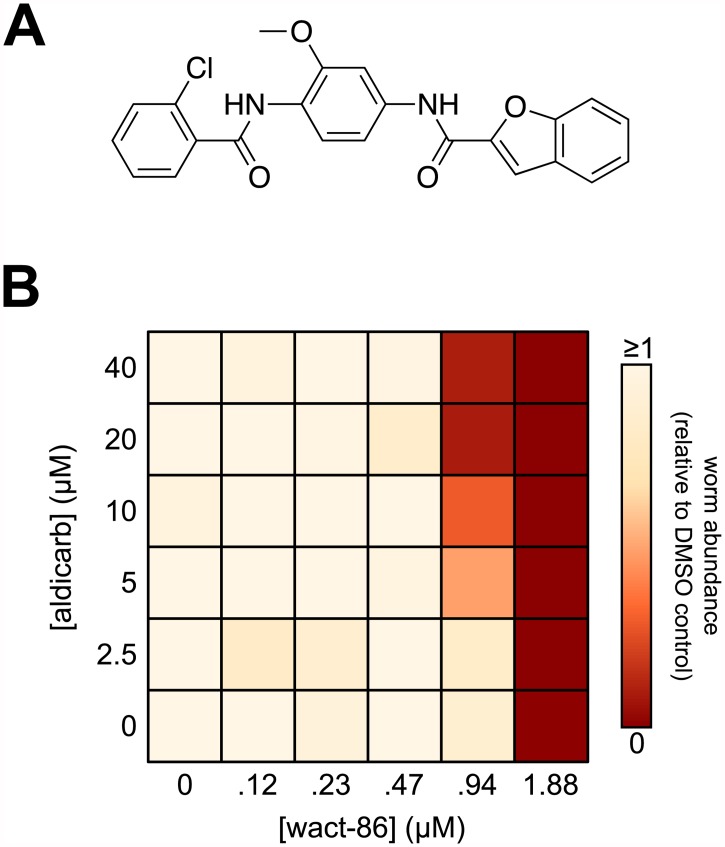
The novel nematicide wact-86 interacts with aldicarb to kill *C*. *elegans*. **(A)** The chemical structure of wact-86. **(B)** Combination dose-response matrix for wact-86 and aldicarb. Worm abundance, relative to the DMSO control, is represented by a colour-coded scale ranging from 0 (no viable worms) to ≥1 (at least as many viable worms as DMSO control). See Methods for how the relative worm abundance value was calculated.

One of the strongest hits we obtained from our screen is wact-86 (N-{4-[(2-chlorobenzoyl)amino]-3-methoxyphenyl}-1-benzofuran-2-carboxamide; see S2 Fig), whose structure is shown in Fig 1A. We re-ordered wact-86 from a commercial source (see Methods), and verified its structure by mass spectrometry (S4 Fig). To validate the interaction between wact-86 and aldicarb we generated a combination dose-response matrix (Fig 1B). We found that maximal interaction is achieved when 20 μM aldicarb and 0.94 μM wact-86 are combined to kill *C*. *elegans* (Fig 1B). At these concentrations neither aldicarb nor wact-86 perturb the growth of worms as single agents (Fig 1B). This result is consistent with our primary screen data, and confirms the interaction between aldicarb and wact-86.

A search of SciFinder’s myriad chemical abstract databases revealed no published abstracts describing worm bioactivity for wact-86, or for any molecule sharing a pairwise structural similarity greater than or equal to 75% with wact-86, suggesting that wact-86 is a novel nematicide with an uncharacterized mechanism-of-action.

### Wact-86 resistant mutants have missense mutations in the *ges-1* gene

**Fig 2:**
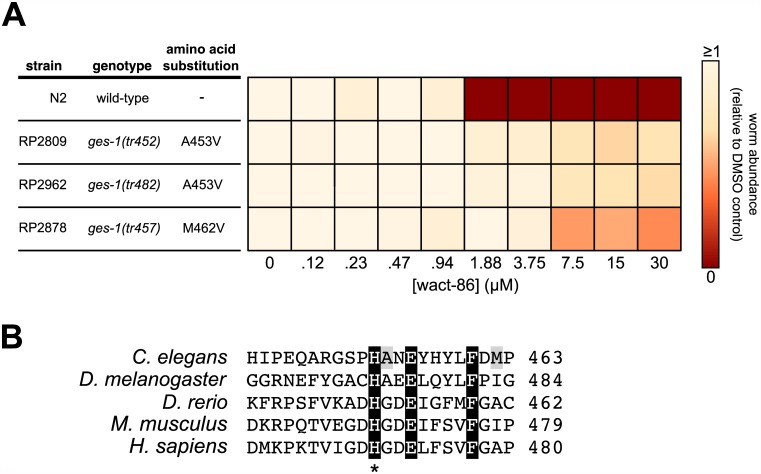
Wact-86 resistant mutants harbour missense mutations in the carboxylesterase gene *ges-1*. **(A)** wact-86 dose-response assays for wild-type worms and the three wact-86 resistant mutants. For each resistant strain the *ges-1* allele and the GES-1 amino acid substitution are indicated (see S2 File for the whole genome sequencing data obtained for the resistant mutants). **(B)** Sequence alignment of the *C*. *elegans* GES-1 protein with the orthologous carboxylesterases from fly, fish, mouse, and human. For clarity, only the segment that is mutated in the wact-86 resistant strains is shown. Conserved residues are highlighted in black. The two GES-1 residues that are mutated in the wact-86 resistant strains are highlighted in grey. The asterisk denotes the conserved histidine that is part of the enzyme’s catalytic triad.

Towards better understanding the mode-of-action of wact-86, and by extension its mode of interaction with aldicarb, we carried out a genetic screen for wact-86 resistant mutants. This type of genetic approach has been used previously to identify the targets and the targeted pathways of bioactive compounds [20,25,28], as well as genes involved in drug detoxification and transport [29,30]. No resistant mutants were isolated in a screen of 100,000 mutagenized genomes in the second filial (F2) generation, suggesting that there are no recessive loss-of-function mutations that are sufficient to confer resistance to wact-86. We also screened 2.8 million mutagenized genomes in the F1 generation and were able to isolate three wact-86 resistant mutant strains (RP2809, RP2878, and RP2962). In contrast to wild-type worms, which are not viable at wact-86 concentrations greater than or equal to 1.88 μM, all three resistant strains display wact-86 resistance up to a concentration of at least 30 μM (Fig 2A).

To identify the wact-86 resistance-conferring mutations we sequenced the genomes of our three resistant strains and found that all three strains harbour missense mutations in the *ges-1* gene (Fig 2 and S2 File). By contrast, no other gene in the genome has a protein-changing substitution in all three strains (S2 File). Furthermore, *ges-1* is not mutated in 56 distinct mutagenized strains obtained from two unrelated genetic screens carried out by our group previously [25,31]. Thus, it is unlikely that *ges-1* would be mutated in all three wact-86-resistant genomes by random chance alone. None of the *ges-1* mutations are nonsense, frame-shifts, or deletions that are indicative of a loss-of-function. Instead, the missense mutations cause an A453V substitution in the RP2809 and RP2962 strains, and a M462V substitution in the RP2878 strain (Fig 2 and S2 File). These observations are consistent with the idea that these mutations confer a dominant gain-of-function phenotype that would be manifested in an F1 screen. Despite having the same missense mutation, RP2809 and RP2962 are clearly independently isolated mutants with distinct background mutations (S2 File). The RP2809 and RP2962 strains have greater wact-86 resistance compared with RP2878, perhaps indicating a correlation between *ges-1* genotype and the wact-86 resistance phenotype (Fig 2A). Taken together, these data suggest that the *ges-1* mutations may confer resistance to wact-86.

*ges-1* encodes a carboxylesterase enzyme that catalyzes the hydrolysis of carboxylic ester bonds [32–34]. The GES-1 enzyme has relatively broad substrate specificity, and is thus classified as a non-specific esterase [32–34]. The expression of *ges-1* is restricted to the intestine, pharynx, and rectum of *C*. *elegans* [32,35,36], but it is responsible for approximately half of the total esterase activity in worms [34]. In humans, orthologous liver carboxylesterases have been implicated in the hydrolysis of a number of drugs including cocaine and heroin [37], thereby facilitating their detoxification. In addition to carboxylic ester hydrolysis, carboxylesterases can also hydrolyse amide bonds, albeit less efficiently [38].

The GES-1 residues that are mutated in the wact-86 resistant mutants are in close proximity to residues that are conserved across phyla (Fig 2B). The high degree of conservation of these residues might implicate them as being important for enzymatic function. For instance, Ala453, which is mutated in two of the wact-86 resistant strains, is immediately C-terminal to His452, which is one of three conserved residues that make up the catalytic triad at the active site of the enzyme [39] (Fig 2B). Given their proximity to such highly conserved and functionally important residues, it is possible that the *ges-1* mutations modify GES-1 activity.

### GES-1-dependent hydrolysis of wact-86 is increased in wact-86 resistant mutants

Wact-86 contains two separate amide bonds that link three distinct aryl groups (Fig 1A). In light of the role carboxylesterases play in human drug metabolism, and given their ability to hydrolyze amide bonds, we hypothesized that the *ges-1* mutations in our resistant mutants are gain-of-function, and that they confer wact-86 resistance by allowing for more efficient hydrolysis and detoxification of wact-86 by the GES-1 enzyme. This hypothesis is consistent with the expression of *ges-1* in the pharynx and intestine, which is likely the point of entry for many xenobiotics into the tissues of the worm.

**Fig 3:**
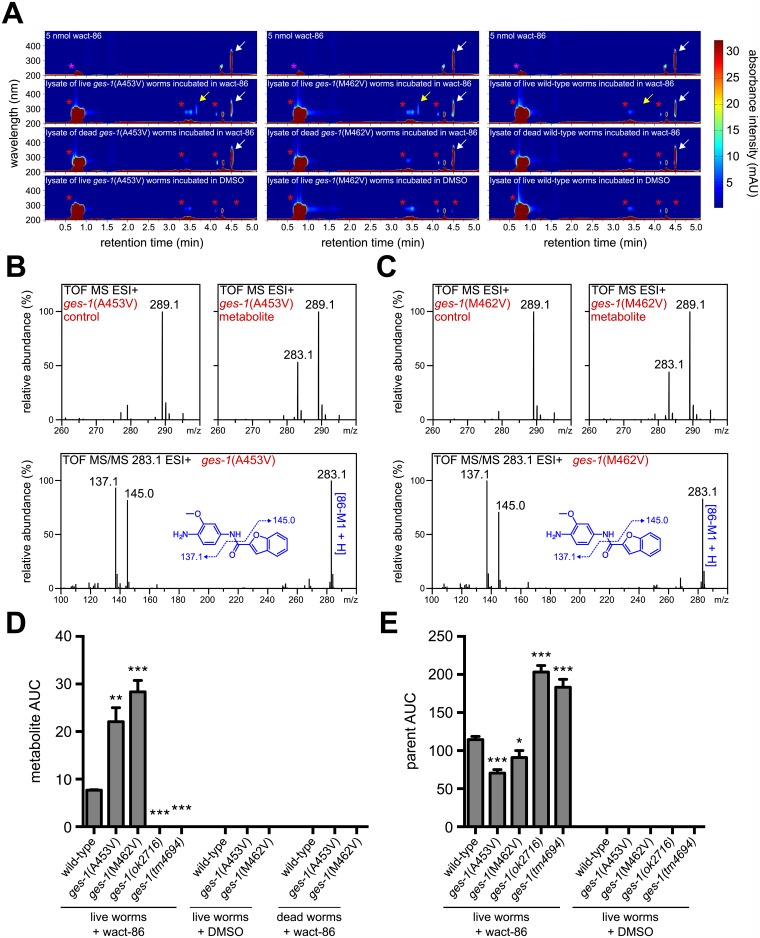
Wact-86-resistant mutants have increased GES-1-dependent hydrolysis of wact-86. **(A)** HPLC analysis of wact-86 metabolism for the indicated genotypes. HPLC-DAD chromatograms for the lysates of live worms incubated in DMSO alone, the lysates of live and dead worms incubated in wact-86, and 5 nmol of wact-86 stock compound are shown. The magenta asterisks indicate the DMSO peak, the red asterisks indicate the peaks of endogenous worm metabolites, the white arrow indicates the wact-86 parent structure peak, and the yellow arrow indicates the presumptive wact-86 metabolite peak. The peak that elutes at ~4.3 minutes in all of the chromatograms is a background instrument peak. **(B and C)** Mass spectral data for the DMSO control and metabolite fractions collected from the lysates of worms with the indicated genotypes, as well as tandem MS/MS fragmentation spectra for the 283.1 mass found exclusively in the metabolite fractions (see S1 Table for accurate mass data). **(D and E)** Quantification of wact-86 metabolite **(D)** and parent structure **(E)** accumulation in worms incubated in 30 μM wact-86. The genotypes are indicated. *ok2716* and *tm4694* are deletion alleles of *ges-1*. For control purposes, quantification was also performed for worms incubated in DMSO alone, as well as for dead worms incubated in wact-86, where appropriate. Area under the curve (AUC) was calculated at a wavelength of 290 nm and a retention time of 3.7 minutes for the metabolite. For the wact-86 parent structure, AUC was calculated at a wavelength of 316 nm and a retention time of 4.5 minutes. One, two, and three asterisks indicate student’s t-test p < 0.05, p < 0.01, and p < 0.001, respectively, compared to wild-type. Error bars represent the s.e.m.

We have previously shown that drug metabolites in worm lysates can be separated, visualized, and quantified using a **h**igh **p**erformance **l**iquid **c**hromatography system coupled with a variable wavelength **d**iode **a**rray **d**etector (HPLC-DAD) [40]. We typically employ reversed-phase HPLC such that metabolites with greater aqueous solubility than the unmodified parent compound will elute earlier from the column than the parent structure (see Methods). To determine whether the wact-86 resistant mutants hydrolyze wact-86, we incubated RP2809, which contains a *ges-1*(A453V) mutation, and RP2878, which contains a *ges-1*(M462V) mutation, in 30 μM wact-86 for 2 hours, after which we lysed the worms and examined the contents of the lysates using our HPLC-DAD system. We identified two absorbance peaks in the wact-86-treated lysates that are absent from the DMSO control lysates (Fig 3A). The two peaks have retention times of 3.7 and 4.5 minutes, and absorbance maxima of 290 and 316 nm, respectively. The 4.5-minute peak likely corresponds to the wact-86 parent structure, since its retention time and absorbance spectrum are identical to the wact-86 standard (Fig 3A). The 3.7-minute peak is not present in the wact-86 standard, and it is absent from the lysates of heat-killed worms incubated in wact-86, suggesting that it may be a *bona fide* metabolite of wact-86 and not merely a wact-86 degradation product (Fig 3A).

To determine the structural identity of the presumptive wact-86 metabolite, we HPLC-purified it from the lysates of *ges-1*(A453V) and *ges-1*(M462V) mutants incubated in wact-86, and analyzed it by mass spectrometry (MS). For control purposes we collected the same HPLC fraction from lysates derived from worms incubated in DMSO alone and performed the same MS analysis. We identified a mass of 283.1 that was present in both of the mutant metabolite fractions, but was absent from both of the DMSO control fractions (Fig 3B and 3C). Hydrolysis of the amide bond that joins the 2-chlorophenyl and the anisole groups in wact-86 would produce two metabolites: *N*-(4-amino-3-methoxyphenyl)benzofuran-2-carboxamide (86-M1) and 2-chlorobenzoic acid (86-M2), which have exact masses of 282.1 and 156, respectively (see S5 Fig). The mass of 283.1 we identified in the mass spectra of our metabolite fractions is consistent with a protonated form of 86-M1. Fragmenting this mass by tandem MS/MS produced two abundant masses of 137.1 and 145.0, consistent with amide bond cleavage of 86-M1 (Fig 3B and 3C). Accurate mass determinations of the 283.1 mass confirm that the metabolite is indeed the wact-86 hydrolysate 86-M1 (S1 Table).

To test the hypothesis that our wact-86 resistant mutants hydrolyze wact-86 more efficiently than wild-type animals, we used our HPLC-DAD system to quantify the abundance of 86-M1 in the lysates of wild-type, *ges-1*(A453V), and *ges-1*(M462V) worms incubated in 30 μM wact-86 for 2 hours. Consistent with our hypothesis, the 86-M1 metabolite is 3 to 4-fold more abundant in the resistant mutants compared to wild-type worms (Fig 3A and 3D). If the resistant mutants metabolize and detoxify wact-86 more efficiently than wild-type animals then the amount of unmodified wact-86 should be greater in wild-type worms relative to the resistant mutants. Indeed, we found that the resistant mutants contain significantly less wact-86 in their tissues compared to wild-type worms (Fig 3E).

To test whether wact-86 hydrolysis depends on the activity of GES-1, we incubated two different *ges-1* deletion mutants in 30 μM wact-86 and analyzed the worm lysates by HPLC-DAD. In contrast to the lysates obtained from wild-type and wact-86 resistant worms, we found that the deletion mutant lysates have no detectable wact-86 metabolite (Fig 3D), suggesting that wact-86 metabolism depends on GES-1 enzymatic activity. Altogether, these data support the idea that GES-1 hydrolyzes wact-86 *in vivo*, and that GES-1 activity is increased in the wact-86 resistant mutants, thus providing a mechanism for resistance.

### Aldicarb interacts with wact-86 by inhibiting its GES-1-dependent hydrolysis

In addition to inhibiting acetylcholinesterase activity, aldicarb is known to inhibit other carboxylesterase enzymes [41], including GES-1 [33]. Thus, one model to explain the interaction between aldicarb and wact-86 is that aldicarb inhibits the GES-1-dependent hydrolysis of wact-86, thereby preventing its detoxification and enhancing its nematicidal activity. This model espouses three predictions: 1) The wact-86 hypersensitivity of the *ges-1* deletion mutants, if they are functionally null for wact-86 hydrolysis, should be similar to that of wild-type worms treated with aldicarb at a concentration affording maximal interaction with wact-86 (i.e. 20 μM aldicarb–see Fig 1B); 2) Aldicarb treatment should not further sensitize the *ges-1* null mutant to wact-86; 3) Aldicarb should inhibit the GES-1-dependent hydrolysis of wact-86 *in vivo*.

**Fig 4:**
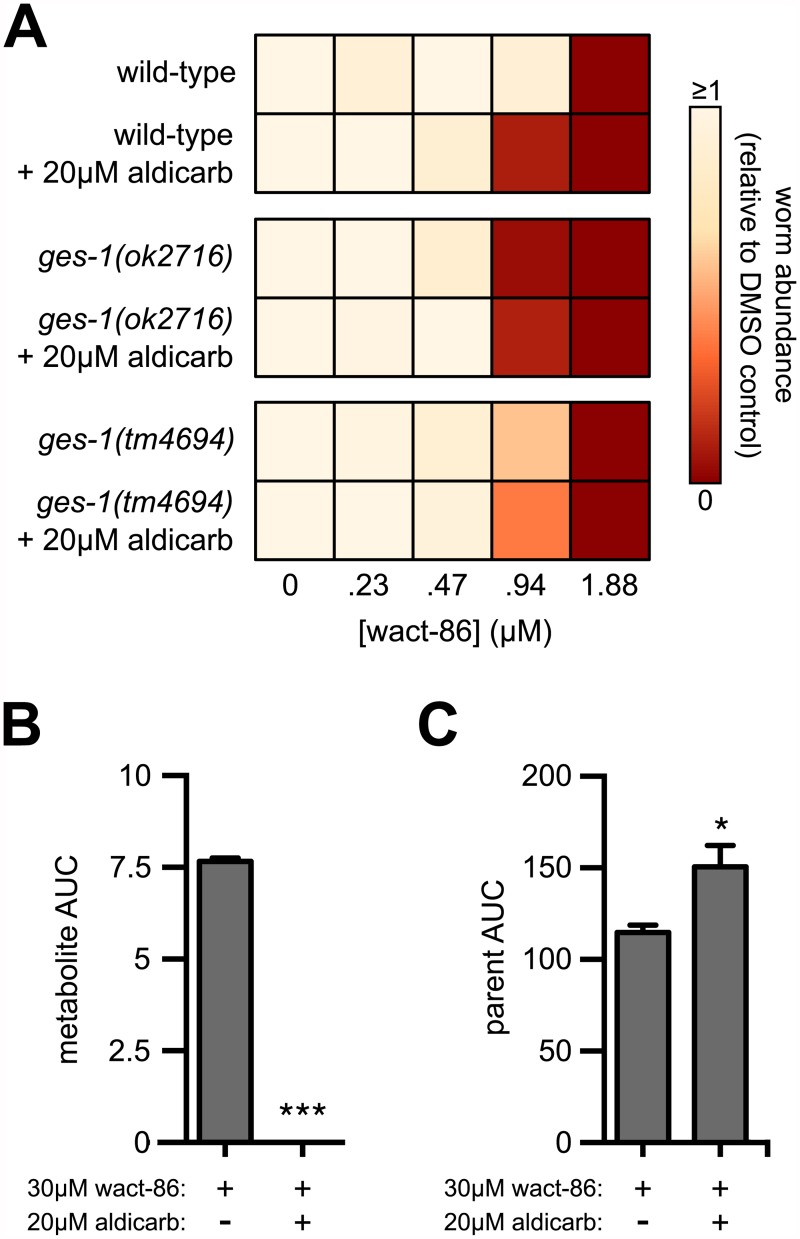
Aldicarb potentiates wact-86 activity by inhibiting its GES-1-dependent hydrolysis. **(A)** wact-86 dose-response assays, plus and minus 20 μM aldicarb, for wild-type worms and two strains harbouring *ges-1* deletion alleles. **(B)** Quantification of wact-86 metabolite accumulation in wild-type worms incubated in wact-86 alone or in combination with aldicarb. **(C)** Quantification of wact-86 accumulation in wild-type worms incubated in wact-86 alone or in combination with aldicarb. For B and C, the area under the curve (AUC) values for the wact-86 parent and metabolite were calculated as in Fig 3. One and three asterisks indicate student’s t-test p < 0.05 and p < 0.001, respectively, compared to the aldicarb-untreated condition. Error bars represent the s.e.m.

In agreement with the first and second predictions, wild-type animals treated with 20 μM aldicarb phenocopy the wact-86 hypersensitivity exhibited by the *ges-1(ok2716)* deletion mutant (Fig 4A), and 20 μM aldicarb does not further sensitize this mutant to wact-86 (Fig 4A). The strain carrying the *tm4694* deletion allele of *ges-1* is also hypersensitive to wact-86, but less so than the strain carrying *ok2716*, suggesting that the *tm4694* allele may retain some hydrolase activity. This result is perhaps not surprising, since the *ok2716* deletion eliminates two residues of the catalytic triad, Ser198 and Glu319, the former being absolutely required for hydrolase activity [39,42], whereas the *tm4694* deletion retains both of these residues (S6 Fig). Depending on its exact location, the *tm4694* deletion may cause a premature stop codon upstream of His452, but the loss of this residue will not necessarily eliminate enzymatic function [43] (S6 Fig). The wact-86 hypersensitivity of the deletion mutants is consistent with these mutants containing 60 to 80% more wact-86 in their tissues relative to wild-type worms (Fig 3E). To test the third prediction, we incubated wild-type worms for two hours in 30 μM wact-86 together with 20 μM aldicarb, and analyzed the lysates using our HPLC-DAD system. Consistent with our prediction, aldicarb treatment inhibits the GES-1-dependent hydrolysis of wact-86, and results in the accumulation of relatively greater amounts of the wact-86 parent compound in worm tissue (Fig 4B). Taken together, our results suggest that aldicarb interacts with wact-86 to kill nematodes by inhibiting its GES-1-dependent hydrolysis and detoxification.

### A subset of the aldicarb interactors interact independently of GES-1

In addition to wact-86, our aldicarb interactor screen yielded 19 distinct compounds that interact positively with aldicarb, seven of which contain either an amide or ester group that could be metabolized by GES-1 (S2 and S3 Figs). To test whether GES-1 inhibition is the likely mechanism of aldicarb interaction for the additional hits, we performed dose-response assays for 13 out of the 19 compounds using wild-type worms, as well as the wact-86 resistant mutant RP2962 and the *ges-1* deletion mutant RB2053 (S7 Fig). We found that the wact-86 resistant mutant is not consistently and robustly resistant to any of the compounds tested, suggesting that it is specifically resistant to wact-86. The *ges-1* deletion mutant is weakly hypersensitive to 9 out of the 13 molecules tested, suggesting that inhibition of GES-1 activity may account for their interaction with aldicarb. Of these nine compounds, five do not contain an amide or ester group, suggesting that these compounds may have an amide or ester group introduced into their structure metabolically before GES-1 can hydrolyze them. For example, hydroxylation of the quinoline C2 carbon of wact-372, followed by enol-keto tautomerism, would reveal a secondary amide which could be hydrolyzed by GES-1, resulting in the opening of the quinoline ring and the potential inactivation of the compound. Regardless, four of the hits are interacting with aldicarb in a *ges-1*-independent manner, suggesting that their mode(s) of interaction are distinct from that of wact-86.

## Discussion

Here we used the free-living nematode *C*. *elegans* to screen for novel chemical interactors of a commercial nematicide. We identified 20 compounds that interact with aldicarb to perturb worm growth, and we characterized the mode of interaction for one of these, wact-86, in detail. Numerous lines of genetic and biochemical evidence show that the interaction between wact-86 and aldicarb derives from aldicarb’s inhibition of GES-1. We have shown that GES-1 hydrolyzes wact-86, which is lethal to *C*. *elegans*. Aldicarb’s inhibition of GES-1 therefore increases the potency by which wact-86 kills worms.

How wact-86 kills *C*. *elegans* remains unknown. Our previous work has shown that it is also able to kill *C*. *briggase*, and has some activity against at least two parasitic nematodes [25]. Exhaustive forward genetic screens for dominant and recessive *C*. *elegans* mutants that resist wact-86 failed to yield its target, suggesting that wact-86’s target is not genetically accessible. Furthermore, chemoinformatic searches using Scifinder Scholar and the Similarity Ensemble Approach online search tool [44] did not reveal any obvious candidate targets. Hence, other approaches will be needed to determine the mechanism by which wact-86 kills *C*. *elegans*.

GES-1 is the predominant esterase expressed in the intestine of *C*. *elegans* and likely has important roles in the metabolism of exogenous molecules and nutrients [34,45]. Because it is largely expressed in the intestinal lineage, it has been used as a marker for *C*. *elegans* gut development for nearly 30 years [32]. Despite its importance in metabolism, *ges-1* mutants lack phenotypes that are obvious at the level of the dissection microscope [34]. However, animals deficient in GES-1 activity are hypersensitive to wact-86, while animals with increased GES-1 activity exhibit resistance to the lethal effects of wact-86. Hence, wact-86 is a new tool that can be exploited to genetically dissect *ges-1* function.

Despite the mild interaction between aldicarb and wact-86, this work provides proof-of-principle that *C*. *elegans* can be a useful platform with which to: i) screen for new molecules that positively interact with known nematicides and, ii) understand the mechanism of their interaction. In addition to wact-86, our screen revealed 19 other compounds that interact with aldicarb, and a subset of these are likely interacting with aldicarb in a distinct, *ges-1*-independent manner. Future work may reveal the nature of these interactions with aldicarb.

## Materials and methods

### Chemical sources

The sources for the chemicals used in the aldicarb interactor screen are indicated in S1 File. For follow-up experiments, wact-86 was purchased from the ChemBridge Corporation and the Vitas-M Laboratory. Wact-86 from both vendors had comparable activity.

### *C*. *elegans* strains and culture methods

The N2 (wild-type) strain of *C*. *elegans* as well as the *ges-1* deletion strain RB2053 were obtained from the Caenorhabditis Genetics Center (University of Minnesota). The strain harbouring the *ges-1(tm4694)* deletion allele was obtained from the Mitani Lab (Tokyo, Japan). All strains were cultured using standard methods [46]. The N2, RP2927, RB2053, and *tm4694*-containing strains were cultured at 20°C. To compensate for their relatively slower growth rates, RP2809 and RP2962 were cultured at room temperature (~22°C).

### Aldicarb chemical interactor screen

The aldicarb interactor screen was carried out in 96-well plates using our previously described *C*. *elegans* liquid-based chemical screening assay [25]. In brief, a saturated culture of HB101 *E*. *coli* was concentrated 2-fold with liquid nematode growth medium (see Ref. [25] for the NGM recipe). 80 μL of NGM+HB101 media was dispensed into the 96-well plate wells, and aldicarb (or DMSO alone for the control screens), was pinned into the wells using a pinning tool with a 300 nL slot volume (V&P Scientific). The wactive library chemicals were then pinned into the wells using the same pinning tool. Approximately 40 synchronized first larval-stage (L1) worms were added to each well in 20 μL of M9 buffer (see Ref. [47] for the M9 recipe). Synchronized L1s were obtained from an embryo preparation (see Ref. [47] for the protocol) performed the previous day. The final DMSO concentration in the wells was 0.6% v/v, the final aldicarb concentration was 10μM, and the final concentration of the wactive compounds was 1.5 μM. The plates were sealed with parafilm, placed upright into a Tupperware box containing many paper towels soaked with water, and then incubated at 20°C with shaking at 200 rpm for 6 days. After the 6-day incubation, a dissection microscope was used to count the number of viable worms in each well. The screen was repeated twice. Hits from the screen were identified as compounds that perturbed worm growth in combination with aldicarb in both replicates, but had no obvious effect on worm development as single agents.

### Aldicarb dose-response assay

The aldicarb dose-response experiments were carried out in 96-well plates using the liquid-based assay we have previously described (see above) [25]. 20 synchronized L1s were added to each well, and incubated for 6 days at 20°C. After 6 days, the number of viable worms in each well was counted, and the relative worm abundance was calculated by dividing the number of viable worms in a given aldicarb-containing well by the number of viable animals in the DMSO control well. Any well with 20 or more viable animals was counted as having twenty viable animals. Four technical replicates were performed, and the relative worm abundance was calculated as an average across the four replicates.

### Dose-response assays on solid media

All of the dose-response experiments, with the exception of the aldicarb dose-response assay described above, were carried out in 24-well plates using a solid-based assay that we have described previously [47]. Briefly, in each well, the desired amount of wact-86 and/or aldicarb was dissolved in 1 mL of molten MYOB + 2% agar media (see Ref. [47] for the recipe), ensuring that the final concentration of DMSO (i.e. the vehicle) was 1% v/v. The plates were left overnight at room temperature to solidify. The following day, the plates were dried for 45 minutes in a sterile laminar flow hood, after which 25 μL of a saturated OP50 culture in LB media was deposited into each well. The plates were again allowed to dry overnight. The next day ~ 50 synchronized L1-stage larvae were added to each well in 10 μL of M9 buffer. The plates were wrapped in parafilm and stored upside down for 3 days at 20°C. On day 3, the number of viable worms in each well was counted. Relative worm abundance was calculated by dividing the number of viable worms in a given well by the number of viable animals in the DMSO control well. The dose-response experiments were performed at least three times, and the average relative worm abundance was calculated across the experimental replicates. Some of the wells had more worms deposited in them relative to the DMSO control, and so they have relative worm abundance values that exceed 1.

### Forward genetic screen and whole genome sequencing

The forward genetic screen for wact-86 resistant mutants was carried out as previously described [20,25,47]. Briefly, wild-type parent (P0) worms were mutagenized with either 50 mM ethyl methanesulfonate (EMS) or 0.5 mM N-ethyl-N-nitrosourea (ENU) for 4 hours. For an individual screen, 100,000 synchronized L1s from the mutagenized F1 progeny were dispensed onto a 10 cm MYOB agar plate (see Ref. [47] for the protocol to make MYOB agar media) containing 50 μM wact-86. In total, 1.4 million mutagenized F1 animals were screened, which is equivalent to 2.8 million haploid genomes. Resistant worms were identified as those that can grow in the presence of the chemical. RP2878 was obtained from an EMS screen. RP2809 and RP2962 were obtained from ENU screens. Whole genome sequencing of the three wact-86 resistant mutants, and subsequent sequence analysis, was carried out as previously described (see Refs. [25] and [47] for a full description of our methods).

### Multiple sequence alignment

The multiple sequence alignment was carried out using Clustal Omega. The *C*. *elegans* sequence was obtained from WormBase (http://www.wormbase.org). All other sequences were obtained from the National Center for Biotechnology Information protein database.

### HPLC analysis

Synchronized hatchlings were obtained from an embryo preparation of gravid adults (see Ref. [47] for the embryo preparation protocol). For the incubations, 60,000 hatchlings in 500 μL of M9 buffer (see Ref. [47] for an M9 buffer recipe) were treated with either 30 μM wact-86, 30 μM wact-86 in combination with 20 μM aldicarb, or DMSO alone for control purposes. The final concentration of DMSO in all samples was 1% v/v. Prior to the incubations, the hatchlings used for the dead worm controls were heat-killed at 37°C without aeration for 24 hours, and then at 95°C for 20 minutes. The incubations were carried out in standard 1.5-mL micro-centrifuge tubes on a nutating shaker, at 20°C for 2 hours. After the 2-hour incubation, the worms were transferred to the wells of a Pall AcroPrep 96-well filter plate (0.45-μm GHP membrane, 1-ml well volume), the buffer was drained from the wells by vacuum, and the worms were subsequently washed three times with 500 μL of M9 buffer. After washing, the worms were re-suspended in 35 μL of M9 buffer, transferred to a new standard 1.5-mL micro-centrifuge tube, and stored frozen at -80°C. The samples were later lysed by adding 35 μL of a 2X lysis solution (100 mM KCl, 20 mM Tris (pH 8.3), 0.4% SDS, 120 μg mL^-1^ proteinase K), and incubating the tubes at 56°C for 1 hour.

Prior to HPLC, 70μL of acetonitrile was added to the lysates. The samples were mixed by vortexing for approximately 10 seconds, and then centrifuged at 17,949*g* for 2 minutes. After centrifugation, 100 μL of the lysate was injected onto a 4.6 X 150 mm Zorbax SB-C8 column (5 micron particle size) and eluted with solvent and flow rate gradients over 5.2 minutes as indicated in Table 1.

**Table 1:** The HPLC solvent and flow rate gradients used herein. Solvent A is 4.9:95:0.1 (ACN:H_2_O:Acetic Acid); Solvent B is 95:4.9:0.1 (ACN: H_2_O:Acetic Acid).

Time (min)	Solvent	Flow Rate (ml/min)
0.00	Solvent A: 85% Solvent B: 15%	1.5
0.15	Solvent A: 85% Solvent B: 15%	2.0
3.20	Solvent A: 30% Solvent B: 70%	2.0
4.25	Solvent B: 100%	3.0

UV-Vis absorbance was measured every 2 nm between 190 and 602 nm. Absorbance intensity data was converted to three-dimensional heat-mapped chromatograms using MATLAB (The MathWorks). Prior to processing the worm lysates, a 5 nmol amount of pure wact-86 was processed by HPLC to determine its elution time and absorbance spectrum. HPLC was performed using an HP 1050 system equipped with an autosampler, vacuum degasser, and variable wavelength diode-array detector. The column was maintained at room temperature (~22°C). HP Chemstation software was used for data acquisition and quantification. Area under the curve (AUC) was calculated using the Chemstation peak integration tool, using default settings. The AUC values plotted in Figs 3 and 4 are an average of at least three experimental replicates.

### Mass spectrometry

To purify the wact-86 metabolite, the HPLC fraction between 3.6 and 3.8 minutes was collected from two separate lysates, combined, and dried using a Genevac EZ-2 centrifugal evaporator. The identical fraction from DMSO control lysates was also collected and dried. The dried fractions were re-suspended in a minimal volume of 1:1 (*v/v*) methanol: 0.1% aqueous formic acid. Electrospray ionization mass spectrometry (ESI-MS) analyses were carried out using a 6538 UHD model quadrupole time-of-flight mass analyzer equipped with an atmospheric pressure ESI source and a 1260 Infinity model HPLC system (Agilent Technologies, Santa Clara, CA). Samples were analyzed via loop injection with mobile phase composed of 1:1 (*v/v*) methanol: 0.1% aqueous formic acid and flowing at a rate of 0.25 mL min^-1^. Mass spectra were recorded in the 2 GHz mode and the high-resolution MS analyses for molecular formula determinations were obtained using external calibration. Tandem MS/MS analyses were obtained via collision-induced dissociation using the targeted MSn function of the acquisition software. MS/MS spectra were recorded sequentially at three different fragmentation voltages (10, 20 and 30 V) and the resulting spectrum was composed of the average of those three collision energies.

## Supporting information

S1 Fig Aldicarb dose-response analysis. Dose-response experiments were performed using wild-type worms. Worm abundance, relative to the DMSO control, is represented by a colour-coded scale ranging from 0 (no viable worms) to ≥1 (at least as many viable worms as DMSO control). See Methods for how the relative worm abundance value was calculated.
(PDF)

S2 Fig Summary of the aldicarb interactor screen data for the 20 hit compounds. The data for two DMSO control replicates and two experimental replicates is summarized with a colour-coded scale of worm growth. A well is considered overgrown if the original larvae added to the well at the outset of the screen grow up to adulthood, lay well over one hundred progeny, and there is no remaining bacteria (i.e. worm food) in the well.
(PDF)

S3 Fig Structures of the 20 hit compounds from the aldicarb interactor screen. (PDF)

S4 Fig Structural validation of the re-stocked wact-86 by mass spectrometry. Mass spectrometry data for wact-86 re-ordered from ChemBridge Corporation **(A)** and the Vitas-M Laboratory **(B)**. The 421.1 mass is consistent with a protonated form of wact-86. **(C)** Accurate mass data for the 421.1 mass.
(PDF)

S5 Fig Putative metabolic products of wact-86 amide bond hydrolysis. The structure of wact-86 is shown, along with the structures of the five possible wact-86 hydrolysates 86-M1, 86-M2, 86-M3, 86-M4, and 86-M5. CLogP and exact mass values are indicated for wact-86 and for each metabolite. CLogP is an estimation of a compound’s hydrophilicity–compounds with relatively higher CLogP values are less hydrophilic than those with relatively lower CLogP values. CLogP and exact mass were calculated using ChemDraw Professional 15.0.
(PDF)

S6 Fig Sequence alignment of the GES-1 protein sequences from wild-type worms and the *ges-1* deletion mutants. For each allele, the GES-1 protein sequence was translated *in silico* from the spliced *ges-1* genomic nucleotide sequence. Deleted residues are indicated with dashed lines. Regions containing residues that differ from the wild-type sequence are underlined. The three residues of the catalytic triad (Ser198, Glu319, and His452) are highlighted in green. The wild-type *ges-1* nucleotide sequence was taken from WormBase (http://www.wormbase.org). Sanger sequencing was used to determine the sequence of the *ges-1(ok2716)* deletion allele, using primers that flank the deletion. The location of the *ges-1(tm4694)* deletion is reported on the website of the National Bioresource Project (http://shigen.nig.ac.jp/c.elegans/). The *tm4694* allele contains a 194 base pair deletion at one of two possible locations, with breakpoints at T2889 and T3084 (tm4694_1) or at A2890 and A3085 (tm4694_2). The two distinct protein sequences for the two possible *tm4694* deletion locations are included in the sequence alignment. Exon boundaries for all of the *ges-1* alleles were determined using HMMgene (v1.1), which can be found at this URL: http://www.cbs.dtu.dk/services/HMMgene/. The sequence alignment was carried out using Clustal Omega.
(PDF)

S7 Fig Dose-response assays for wact-86 and 13 of the additional aldicarb-interacting screening hits. Dose-response experiments were performed for wild-type worms, the wact-86 resistant mutant RP2962, and the *ges-1* deletion mutant RB2053. For each strain the *ges-1* allele and the GES-1 amino acid substitution are indicated. The structure for each compound is shown to the right of the heat-mapped dose-response assays.
(PDF)

S1 Table Accurate mass data for the wact-86 metabolite 86-M1. (DOCX)

S1 File Aldicarb interactor screen raw data. (XLSX)

S2 File Whole genome sequencing data. (XLSX)
