# Supplementary figures and images for: The novel nematicide wact-86 interacts with aldicarb to kill nematodes
Source: PLoS Negl Trop Dis. 2017 Apr 5;11(4):e0005502. doi: 10.1371/journal.pntd.0005502 (PMC5393889; doi:10.1371/journal.pntd.0005502)

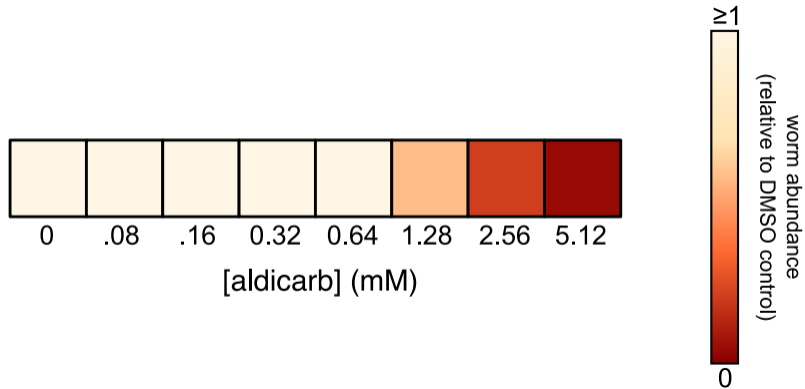

Supplement: S1 Fig — Dose-response experiments were performed using wild-type worms. Worm abundance, relative to the DMSO control, is represented by a colour-coded scale ranging from 0 (no viable worms) to ≥1 (at least as many viable worms as DMSO control). See Methods for how the relative worm abundance value was calculated. (PDF) [file pntd.0005502.s001.pdf]

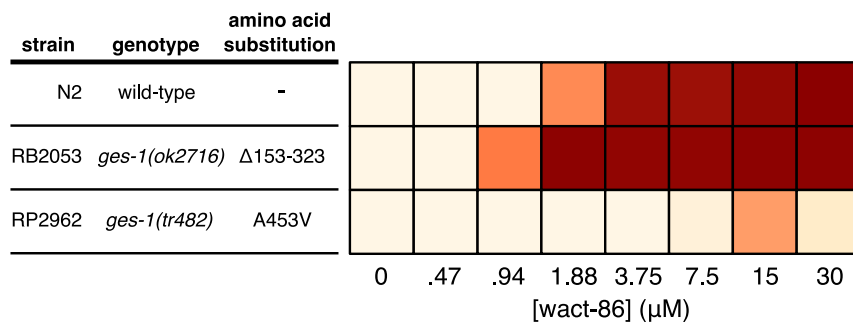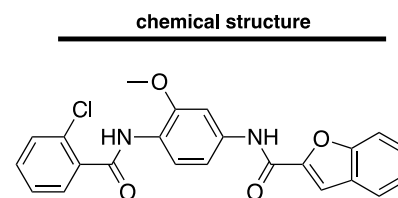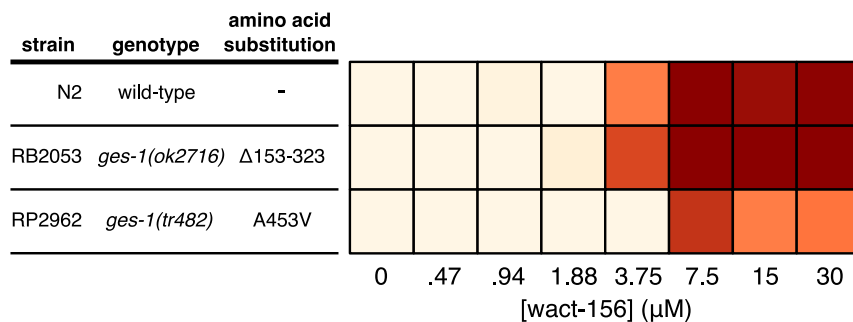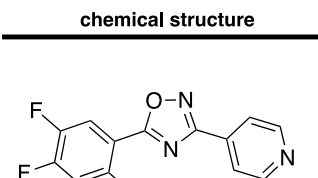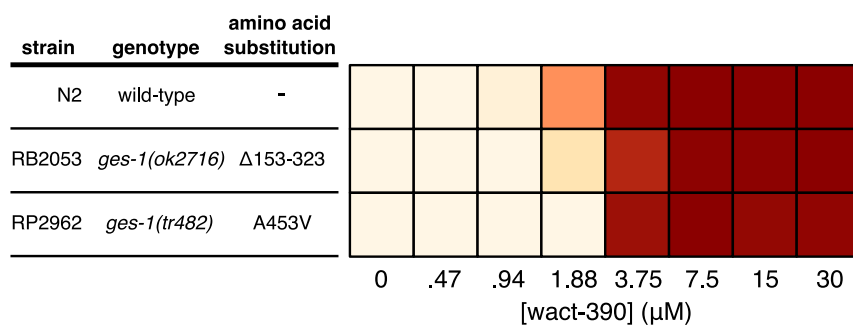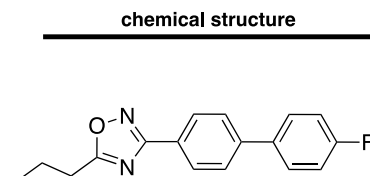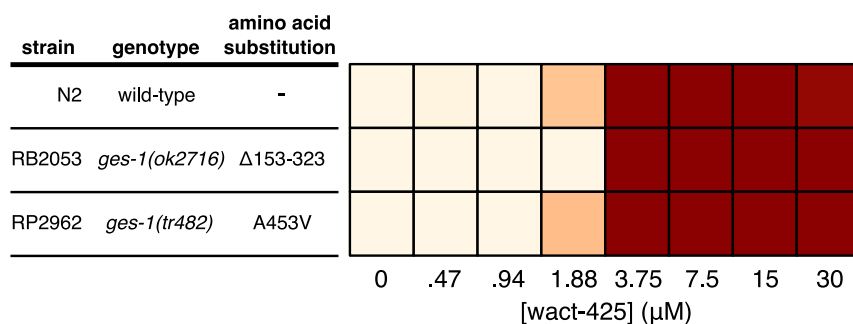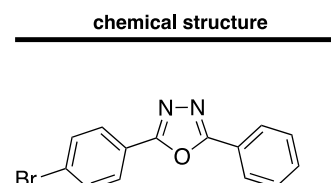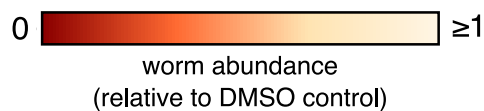

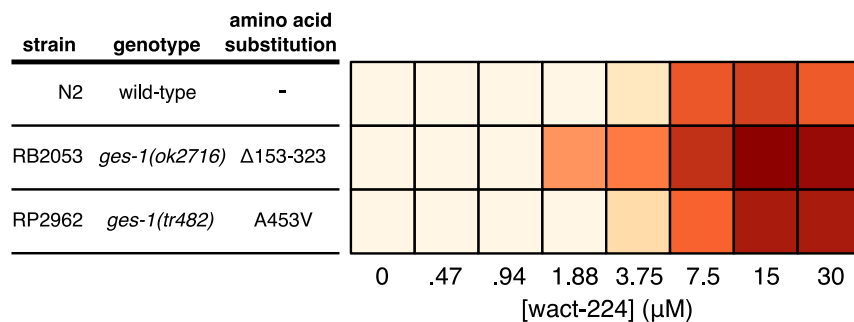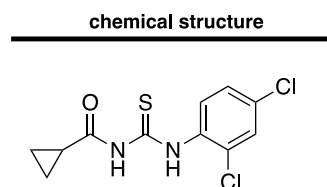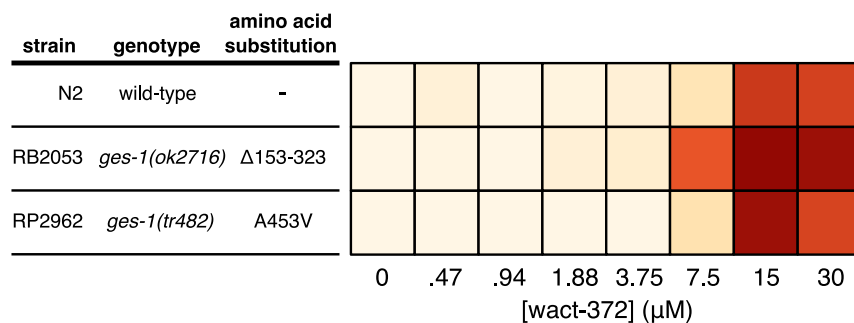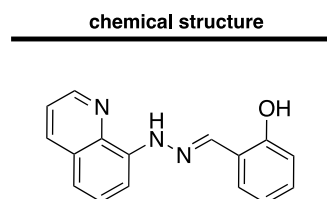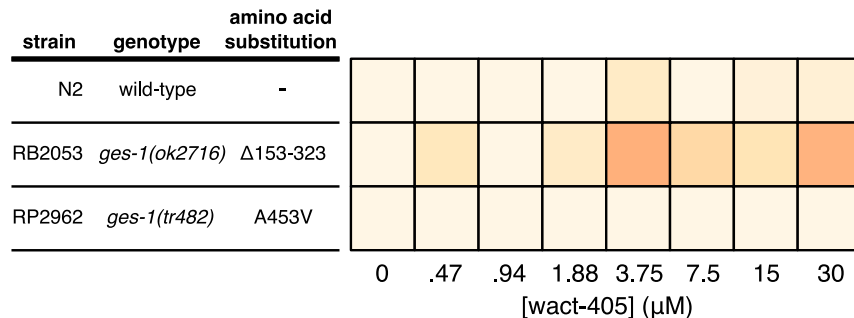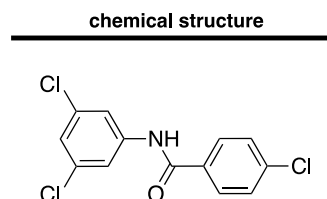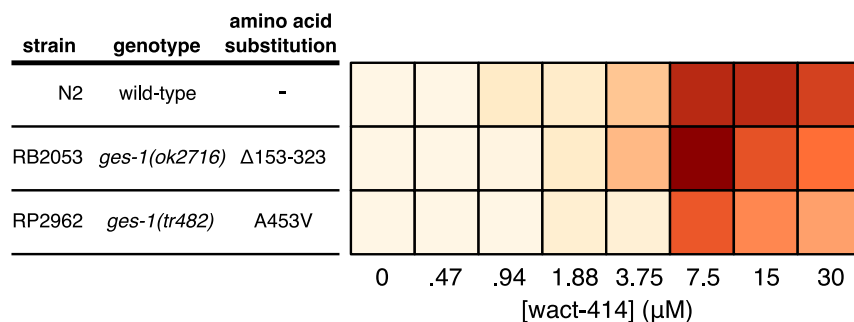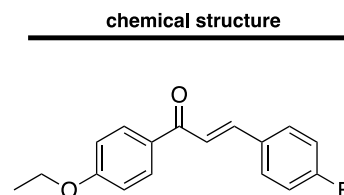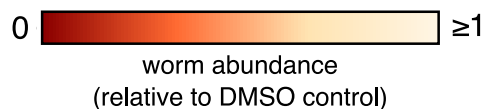

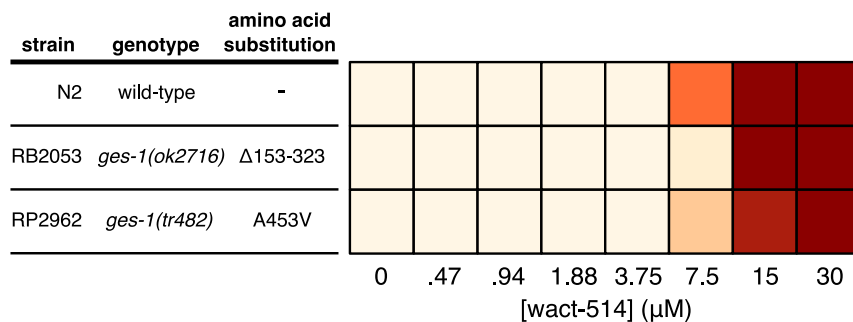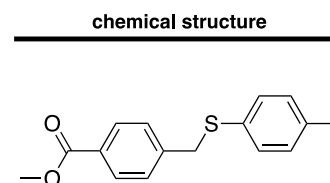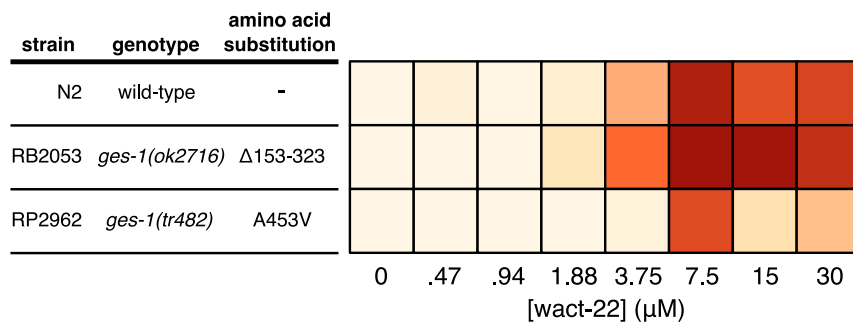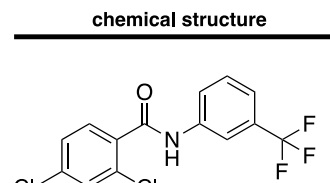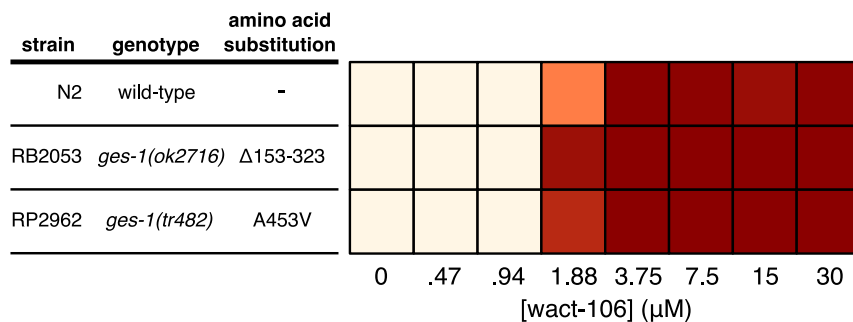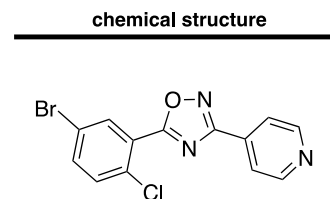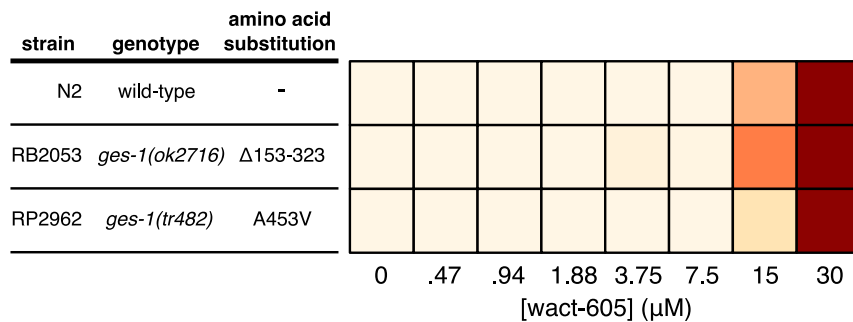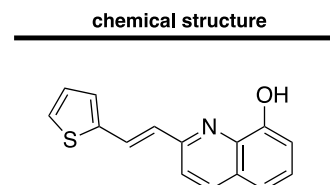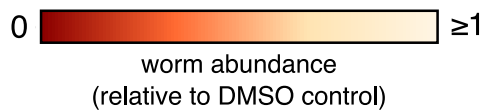

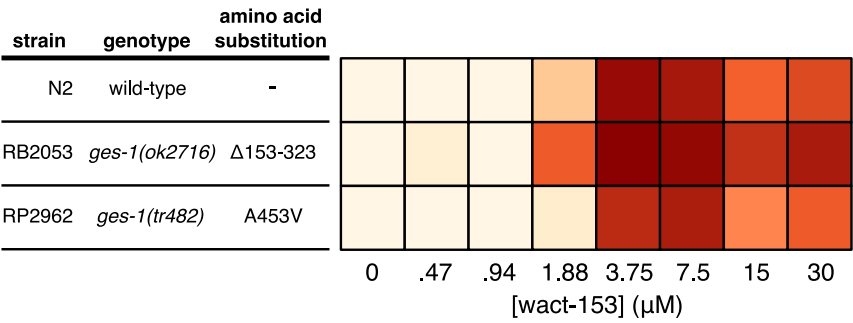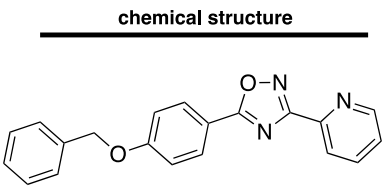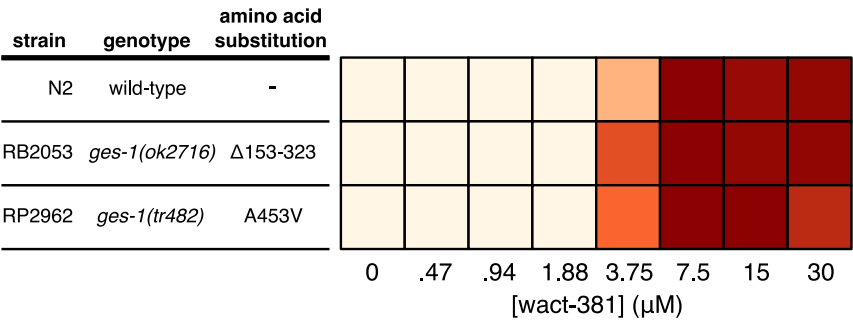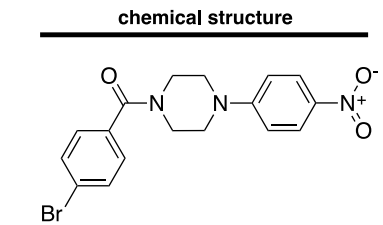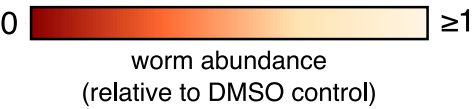

Supplement: S7 Fig — Dose-response experiments were performed for wild-type worms, the wact-86 resistant mutant RP2962, and the ges-1 deletion mutant RB2053. For each strain the ges-1 allele and the GES-1 amino acid substitution are indicated. The structure for each compound is shown to the right of the heat-mapped dose-response assays. (PDF) [file pntd.0005502.s007.pdf]
