# Supplementary material for: The novel nematicide wact-86 interacts with aldicarb to kill nematodes
Source: PLoS Negl Trop Dis. 2017 Apr 5;11(4):e0005502. doi: 10.1371/journal.pntd.0005502 (PMC5393889; doi:10.1371/journal.pntd.0005502)
Supplement: S2 Fig — The data for two DMSO control replicates and two experimental replicates is summarized with a colour-coded scale of worm growth. A well is considered overgrown if the original larvae added to the well at the outset of the screen grow up to adulthood, lay well over one hundred progeny, and there is no remaining bacteria (i.e. worm food) in the well. (PDF) [file pntd.0005502.s002.pdf]

wact ID	DMSO control replicate 1	DMSO control replicate 2	10µM aldicarb replicate 1	10µM aldicarb replicate 2
wact-86				
wact-156				
wact-390				
wact-425				
wact-446				
wact-224				
wact-372				
wact-405				
wact-414				
wact-514				
wact-22				
wact-106				
wact-605				
wact-153				
wact-381				
wact-445				
wact-632				
wact-61				
wact-393				
wact-134				
aldicarb				
aldicarb				
DMSO ctrl				
DMSO ctrl				

Overgrown

More than 50 worms

10 to 50 worms

Less than 10 worms
