# Supplementary material for: The novel nematicide wact-86 interacts with aldicarb to kill nematodes
Source: PLoS Negl Trop Dis. 2017 Apr 5;11(4):e0005502. doi: 10.1371/journal.pntd.0005502 (PMC5393889; doi:10.1371/journal.pntd.0005502)
Supplement: S3 Fig — (PDF) [file pntd.0005502.s003.pdf]

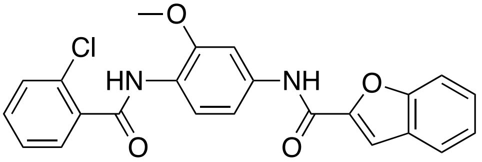

wact-86

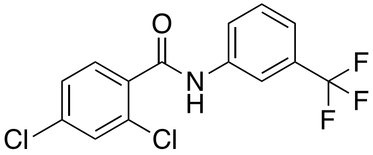

wact-22

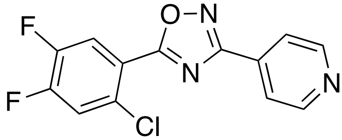

wact-156

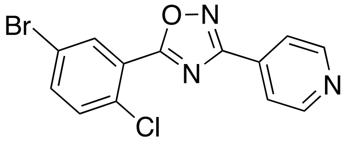

wact-106

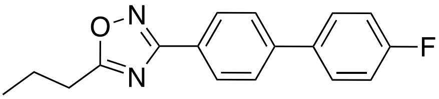

wact-390

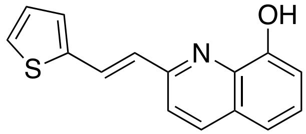

wact-605

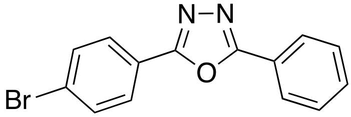

wact-425

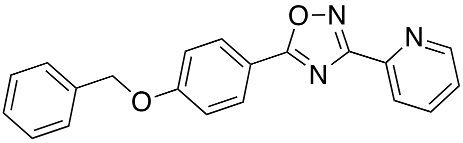

wact-153

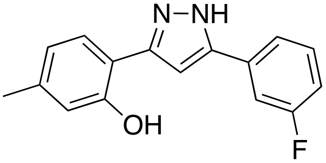

wact-446

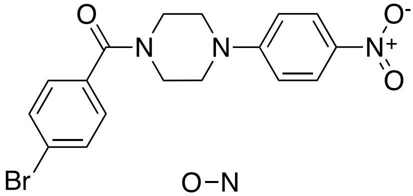

wact-381

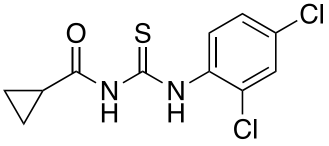

wact-224

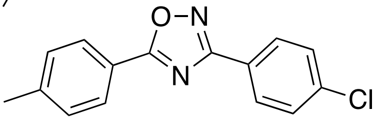

wact-445

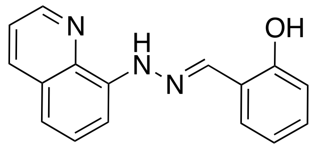

wact-372

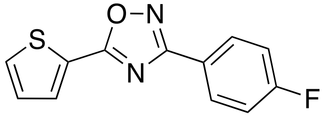

wact-632

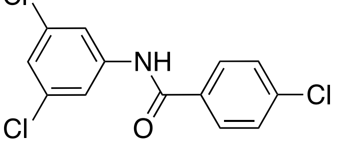

wact-405

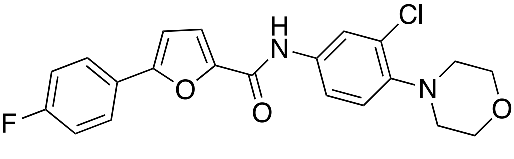

wact-61

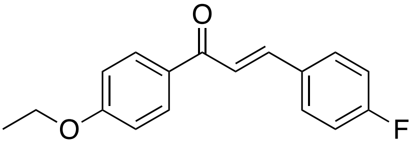

wact-414

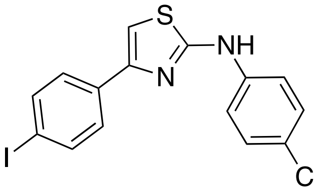

wact-393

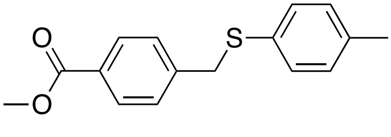

wact-514

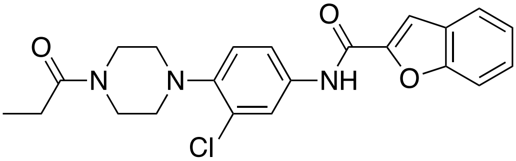

wact-134
