# Supplementary material for: The novel nematicide wact-86 interacts with aldicarb to kill nematodes
Source: PLoS Negl Trop Dis. 2017 Apr 5;11(4):e0005502. doi: 10.1371/journal.pntd.0005502 (PMC5393889; doi:10.1371/journal.pntd.0005502)
Supplement: S4 Fig — Mass spectrometry data for wact-86 re-ordered from ChemBridge Corporation (A) and the Vitas-M Laboratory (B). The 421.1 mass is consistent with a protonated form of wact-86. (C) Accurate mass data for the 421.1 mass. (PDF) [file pntd.0005502.s004.pdf]

A

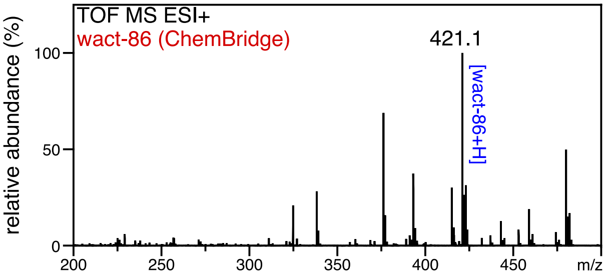

B

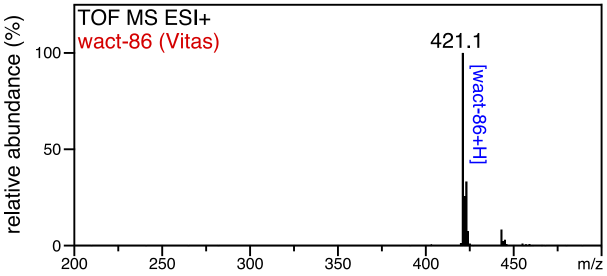

C

Vendor	Target Ion Species	Ionic Formula	Target m/z	Calculated m/z	+/- (mDa)	+/- (ppm)
ChemBridge	$[wact\text{-}86+H]^+$	$C_{23}H_{18}ClN_2O_4$	421.0950	421.0950	0.0	0.0
Vitas	$[wact\text{-}86+H]^+$	$C_{23}H_{18}ClN_2O_4$	421.0957	421.0950	0.7	1.7
