# Supplementary material for: The novel nematicide wact-86 interacts with aldicarb to kill nematodes
Source: PLoS Negl Trop Dis. 2017 Apr 5;11(4):e0005502. doi: 10.1371/journal.pntd.0005502 (PMC5393889; doi:10.1371/journal.pntd.0005502)
Supplement: S5 Fig — The structure of wact-86 is shown, along with the structures of the five possible wact-86 hydrolysates 86-M1, 86-M2, 86-M3, 86-M4, and 86-M5. CLogP and exact mass values are indicated for wact-86 and for each metabolite. CLogP is an estimation of a compound’s hydrophilicity–compounds with relatively higher CLogP values are less hydrophilic than those with relatively lower CLogP values. CLogP and exact mass were calculated using ChemDraw Professional 15.0. (PDF) [file pntd.0005502.s005.pdf]

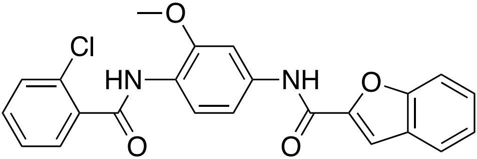

wact-86
CLogP = 3.304
exact mass = 420.1

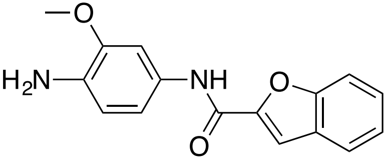

86-M1
CLogP = 2.299
exact mass = 282.1

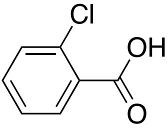

86-M2
CLogP = 2.096
exact mass = 156.0

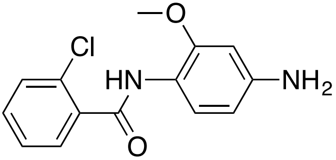

86-M3
CLogP = 1.023
exact mass = 276.1

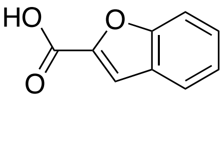

86-M4
CLogP = 2.445
exact mass = 162.0

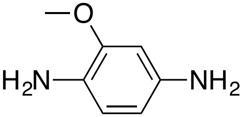

86-M5
CLogP = 0.008
exact mass = 138.1
