# Supplementary material for: The novel nematicide wact-86 interacts with aldicarb to kill nematodes
Source: PLoS Negl Trop Dis. 2017 Apr 5;11(4):e0005502. doi: 10.1371/journal.pntd.0005502 (PMC5393889; doi:10.1371/journal.pntd.0005502)
Supplement: S6 Fig — For each allele, the GES-1 protein sequence was translated in silico from the spliced ges-1 genomic nucleotide sequence. Deleted residues are indicated with dashed lines. Regions containing residues that differ from the wild-type sequence are underlined. The three residues of the catalytic triad (Ser198, Glu319, and His452) are highlighted in green. The wild-type ges-1 nucleotide sequence was taken from WormBase (http://www.wormbase.org). Sanger sequencing was used to determine the sequence of the ges-1(ok2716) deletion allele, using primers that flank the deletion. The location of the ges-1(tm4694) deletion is reported on the website of the National Bioresource Project (http://shigen.nig.ac.jp/c.elegans/). The tm4694 allele contains a 194 base pair deletion at one of two possible locations, with breakpoints at T2889 and T3084 (tm4694_1) or at A2890 and A3085 (tm4694_2). The two distinct protein sequences for the two possible tm4694 deletion locations are included in the sequence alignment. Exon boundaries for all of the ges-1 alleles were determined using HMMgene (v1.1), which can be found at this URL: http://www.cbs.dtu.dk/services/HMMgene/. The sequence alignment was carried out using Clustal Omega. (PDF) [file pntd.0005502.s006.pdf]

```
GES-1_wild-type     MRIFLVSVILINACWAGPIVETNYGKVEGIEYEGAEVFLAIPFAKPPVDDLRFEKPVAPD 60
GES-1_ok2716        MRIFLVSVILINACWAGPIVETNYGKVEGIEYEGAEVFLAIPFAKPPVDDLRFEKPVAPD 60
GES-1_tm4694_1      MRIFLVSVILINACWAGPIVETNYGKVEGIEYEGAEVFLAIPFAKPPVDDLRFEKPVAPD 60
GES-1_tm4694_2      MRIFLVSVILINACWAGPIVETNYGKVEGIEYEGAEVFLAIPFAKPPVDDLRFEKPVAPD 60

GES-1_wild-type     PWEDVYPATQYRNDCTPHYRLVAQFSSYSGEDCLTLNIIKPKKAEKLPVLFWIHGGGYEI 120
GES-1_ok2716        PWEDVYPATQYRNDCTPHYRLVAQFSSYSGEDCLTLNIIKPKKAEKLPVLFWIHGGGYEI 120
GES-1_tm4694_1      PWEDVYPATQYRNDCTPHYRLVAQFSSYSGEDCLTLNIIKPKKAEKLPVLFWIHGGGYEI 120
GES-1_tm4694_2      PWEDVYPATQYRNDCTPHYRLVAQFSSYSGEDCLTLNIIKPKKAEKLPVLFWIHGGGYEI 120

GES-1_wild-type     GSASQHGYEFFAKRYASQGVIVATVQYRLGFMGFFSEGTSDVQGNWGLFDQAAALEFVKS 180
GES-1_ok2716        GSASQHGYEFFAKRYASQGVIVATVQYRLGFM---------------------------- 152
GES-1_tm4694_1      GSASQHGYEFFAKRYASQGVIVATVQYRLGFMGFFSEGTSDVQGNWGLFDQAAALEFVKS 180
GES-1_tm4694_2      GSASQHGYEFFAKRYASQGVIVATVQYRLGFMGFFSEGTSDVQGNWGLFDQAAALEFVKS 180

GES-1_wild-type     NIENFGGDPNQITIWGYSAGAASVSQLTMSPYTRDSYSKAIIMSASSFVGWATGPNVVET 240
GES-1_ok2716        ------------------------------------------------------------ 152
GES-1_tm4694_1      NIENFGGDPNQITIWGYSAGAASVSQLTMSPYTRDSYSKAIIMSASSFVGWATGPNVVET 240
GES-1_tm4694_2      NIENFGGDPNQITIWGYSAGAASVSQLTMSPYTRDSYSKAIIMSASSFVGWATGPNVVET 240

GES-1_wild-type     SKQLAEILGCPWPGAKECMKKKSLHEIFDAIEVQGWTTGTIDILRWSPVIDGDFMTKNPE 300
GES-1_ok2716        ------------------------------------------------------------ 152
GES-1_tm4694_1      SKQLAEILGCPWPGAKECMKKKSLHEIFDAIEVQGWTTGTIDILRWSPVIDGDFMTKNPE 300
GES-1_tm4694_2      SKQLAEILGCPWPGAKECMKKKSLHEIFDAIEVQGWTTGTIDILRWSPVIDGDFMTKNPE 300

GES-1_wild-type     ELIKESPVKPTLIGMSNKEGSYFAALNMGRVIADFGLSPEDMPKVDEEFISEIIGRKLLY 360
GES-1_ok2716        -----------------------AALNMGRVIADFGLSPEDMPKVDEEFISEIIGRKLLY 189
GES-1_tm4694_1      ELIKESPVKPTLIGMSNKEGSYFAALNMGRVIADFGLSPEDMPKVDEEFISEIIGRKLLY 360
GES-1_tm4694_2      ELIKESPVKPTLIGMSNKEGSYFAALNMGRVIADFGLSPEDMPKVDEEFISEIIGRKLLY 360

GES-1_wild-type     NNRYGENREKVWNDILDFYVKQGKPAEVKDLNGFYVDRYSELLSDITFNVPILREITSRV 420
GES-1_ok2716        NNRYGENREKVWNDILDFYVKQGKPAEVKDLNGFYVDRYSELLSDITFNVPILREITSRV 249
GES-1_tm4694_1      NNRYGENREKVWNDILDFYSKQEVKFNWYSSKSLNL------------------------ 396
GES-1_tm4694_2      NNRYGENREKVWNDILDFYASKR------------------------------------- 383

GES-1_wild-type     ERKTPVWTYRMDHYDKNIWKKHIPEQARGSPHANEYHYLFDMPVMAKIDMKKEPDSWIQN 480
GES-1_ok2716        ERKTPVWTYRMDHYDKNIWKKHIPEQARGSPHANEYHYLFDMPVMAKIDMKKEPDSWIQN 309
GES-1_tm4694_1      ----------------------------GSPHANEYHYLFDMPVMAKIDMKKEPDSWIQN 428
GES-1_tm4694_2      ------------------------------------------------------------ 383

GES-1_wild-type     DLIDMVISFAKTGVPQIEDVEWRPVSDPDDVNFLNIRSDGVSIEHGLFQEPLAFWNELRQ 540
GES-1_ok2716        DLIDMVISFAKTGVPQIEDVEWRPVSDPDDVNFLNIRSDGVSIEHGLFQEPLAFWNELRQ 369
GES-1_tm4694_1      DLIDMVISFAKTGVPQIEDVEWRPVSDPDDVNFLNIRSDGVSIEHGLFQEPLAFWNELRQ 488
GES-1_tm4694_2      ------------------------------------------------------------ 383

GES-1_wild-type     REGFDLIDPTNSAMHSSNKDEL 562
GES-1_ok2716        REGFDLIDPTNSAMHSSNKDEL 391
GES-1_tm4694_1      REGFDLIDPTNSAMHSSNKDEL 510
GES-1_tm4694_2      ---------------------- 383
```
