# Supplementary material for: The novel nematicide wact-86 interacts with aldicarb to kill nematodes
Source: PLoS Negl Trop Dis. 2017 Apr 5;11(4):e0005502. doi: 10.1371/journal.pntd.0005502 (PMC5393889; doi:10.1371/journal.pntd.0005502)
Supplement: S1 Table — (DOCX) [file pntd.0005502.s008.docx]

| Strain | Target Ion Species | Ionic Formula | Target  m/z | Calculated  m/z | +/-  (mDa) | +/-  (ppm) |
| --- | --- | --- | --- | --- | --- | --- |
| RP2809 | [86-M1+H]^+^ | C_16_H_15_N_2_O_3_ | 283.1072 | 283.1077 | -0.5 | -1.8 |
| RP2878 | [86-M1+H]^+^ | C_16_H_15_N_2_O_3_ | 283.1080 | 283.1077 | 0.3 | 1.1 |

**S1 Table: Accurate mass data for the wact-86 metabolite 86-M1**
